# A Contribution to the Experimental Diagnosis of Glanders

**Published:** 1884-01

**Authors:** Rudolf Monkeutiu


A Contribution to the Experimental Diagnosis of Glanders.—By Rudolf Monkentin. (Vorträge für Thierärzte, vol. 6, Nos. 2 and 3.

The author begins his essay by a short sketch of the different experimental methods in which investigators have endeavored to prove the existance of glanders in horses by inoculative experiments upon other animals.

Then follows a large number of experiments made by himself upon rabbits and dogs from which he draws the following conclusions.

"From the inocculations which I have made upon rabbits, is is evident that they possess more or less immunity from the action of the infection of glanders.

They are, however, very sensitive to the action of wounds and subcutaneous injections of purulent material proceeding from phlymonia, pyaemia septicaemia, etc.

Inoculations with material taken from horses having glanders frequently produce the above named complications. In some cases we notice the development of rodent ulcerations and nasal glanders, but seldom complication of the internal mucosæ. Sometimes no disease phenomena follow the inoculations. Hence we are justified in concluding: *that rabbits are unsuitable animals upon which to experiment in order to authenticate the diagnosis of glanders in doubtful cases.*

*Each inoculation in dogs was followed by some positive result.* Some displayed small rodent ulcerations with lardaceous bases in the cutis and marasmus in connection with glanders. Death was caused by metastasis to the internal organs and constitutional glanders. Where the inoculated diseases seemed to be localized it was characterized by the formation of ulcerations which finally healed. Inoculations in young dogs gave the best results, and they may be considered as fit subjects for the purposes of substantiating the diagnosis of glanders.

